# Crosstalks between integrin alpha 5 and IGF2/IGFBP2 signalling trigger human bone marrow-derived mesenchymal stromal osteogenic differentiation

**DOI:** 10.1186/1471-2121-11-44

**Published:** 2010-06-23

**Authors:** Zahia Hamidouche, Olivia Fromigué, Jochen Ringe, Thomas Häupl, Pierre J Marie

**Affiliations:** 1 Laboratory of Osteoblast Biology and Pathology, INSERM U606, Paris, F-75475, France; 2 University Paris Diderot, UMR606, Paris, F-75475, France; 3 Rheumatology, Charite University, Berlin, Germany

## Abstract

**Background:**

The potential of mesenchymal stromal cells (MSCs) to differentiate into functional bone forming cells provides an important tool for bone regeneration. The identification of factors that trigger osteoblast differentiation in MSCs is therefore critical to promote the osteogenic potential of human MSCs. In this study, we used microarray analysis to identify signalling molecules that promote osteogenic differentiation in human bone marrow stroma derived MSCs.

**Results:**

Microarray analysis and validation experiments showed that the expression of IGF2 and IGFBP2 was increased together with integrin alpha5 (ITGA5) during dexamethasone-induced osteoblast differentiation in human MSCs. This effect was functional since we found that IGF2 and IGFBP2 enhanced the expression of osteoblast phenotypic markers and *in vitro *osteogenic capacity of hMSCs. Interestingly, we showed that downregulation of endogenous ITGA5 using specific shRNA decreased IGF2 and IGFBP2 expression in hMSCs. Conversely, ITGA5 overexpression upregulated IGF2 and IGFBP2 expression in hMSCs, which indicates tight crosstalks between these molecules. Consistent with this concept, activation of endogenous ITGA5 using a specific antibody that primes the integrin, or a peptide that specifically activates ITGA5 increased IGF2 and IGFBP2 expression in hMSCs. Finally, we showed that pharmacological inhibition of FAK/ERK1/2-MAPKs or PI3K signalling pathways that are enhanced by ITGA5 activation, blunted IGF2 and IGFBP2 expression in hMSCs.

**Conclusion:**

The results show that ITGA5 is a key mediator of IGF2 and IGFBP2 expression that promotes osteoblast differentiation in human MSCs, and reveal that crosstalks between ITGA5 and IGF2/IGFBP2 signalling are important mechanisms that trigger osteogenic differentiation in human bone marrow derived mesenchymal stromal cells.

## Background

Mesenchymal stromal cells (MSCs) can differentiate into chondroblasts, adipocytes or osteoblasts under appropriate stimulation [1-3]. Adult human MSCs (hMSCs) are therefore an important source for tissue repair and therapy in regenerative medicine [4,5]. Expanding the osteogenic capacity of hMSCs is thus of major interest for improving the osteogenic potential of hMSCs for optimal bone regeneration [6]. The osteogenic differentiation of MSCs is characterized by the expression of timely expressed genes such as Runx2, alkaline phosphatase (ALP) and type I collagen (Col1A1) followed by extracellular matrix mineralization [7,8]. Several hormonal and local factors were shown to promote the osteogenic differentiation of MSCs *in vitro*. Notably, short term treatment with dexamethasone was found to induce the expression of osteoblast phenotypic genes in MSCs [9,10]. Unfortunately, glucocorticoids cannot be use therapeutically to promote MSC differentiation because of their long term negative impact on osteoblast recruitment and function [11]. Therefore, identifying genes that are induced by dexamethasone and are involved in the osteogenic differentiation of human MSCs may help to promote their ability to differentiate into cells of the osteoblast lineage.

Here, we used microarray analysis to investigate the molecular mechanisms underlying MSC osteoblast differentiation and enhance the osteogenic potential of human MSCs. We report here that dexamethasone induces the expression of IGF2 and IGFBP2 and show that this effect triggers osteoblast differentiation in human MSCs. We also show that this effect is mediated by integrin alpha5 (ITGA5) expression and signalling. Our data reveal that crosstalks between these signalling molecules are important mechanisms that trigger osteogenic differentiation of human bone marrow derived mesenchymal stromal cells.

## Results

### IGF2/IGFBP2 Expression is Upregulated During Osteoblast Differentiation of MSCs

**Figure 1:**
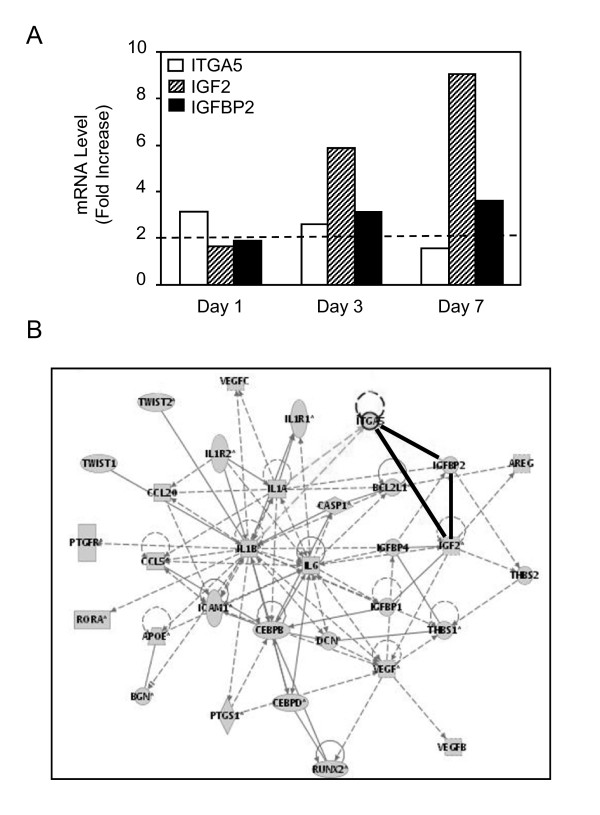
**IGF2 and IGFBP2 Expression is Up-regulated During Osteoblast Differentiation of hMSCs**. Adult hMSCs were treated with or without dexamethasone (10^-7^M) to induce osteoblastic differentiation. Total RNA was collected from three different donors and used for microarray hybridization. Data were normalized and genes that were upregulated (fold change >2) in dexamethasone-treated compared to untreated MSCs were subselected **(A)**. Analysis of gene network using Ingenuity Software indicated interactions between IGF2, IGFBP2 and ITGA5 **(B)**.

**Figure 2:**
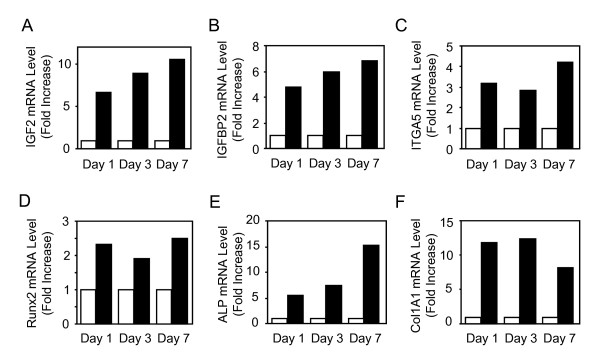
**Coordinated Upregulation of IGF2, IGFBP2 and ITGA5 During Osteoblast Differentiation in hMSCs**. Adult hMSCs were treated with (black boxes) or without (white boxes) dexamethasone (10^-7^M) to induce osteoblastic differentiation and total RNA was analysed using TaqMan Low-Density Array. IGF2, IGFBP2 and ITGA5 mRNA levels were increased by more than 2-fold in dexamethasone-treated MSCs compared to control cells (**A-C**). This was associated with increased expression of the osteoblast phenotypic genes Runx2, ALP and Col1A1 (**D-F**). Results are expressed as fold increase compared to untreated cells, after normalization to 18 S expression. The data are representative of two separate experiments using mRNA from two different donors.

Short term treatment with dexamethasone is known to promote osteogenic differentiation in primary hMSCs [9,10]. Using this differentiating system, we performed a microarray analysis in order to gain insights into the genes that mediate the promoting effect of dexamethasone on MSC osteoblast differentiation. To this goal, total RNA from dexamethasone-treated and untreated MSCs were collected in three different hMSC cultures and used for microarray hybridizations using HG-U133 Plus 2.0 arrays consisting of 54,675 probesets, as described in our original study [11]. Analysis of the normalized microarray data revealed that there were 62, 109 and 84 probe sets identified when selected for a fold change >2 in dexamethasone-treated MSCs compared to control MSCs at 1, 3 and 7 days, respectively. In this follow up study, a sub-selection was made based on the common expression obtained from three different donors and networks identified by the Ingenuity Software. The analysis of normalized data in the three studies showed that two related molecules, namely IGF2 and IGFBP2, as well as ITGA5 were commonly upregulated in dexamethasone-treated MSCs compared to control MSCs at days 3 and 7 (Fig. 1A). IGF2 and IGFBP2, but not ITGA5 remained upregulated at 14 days in dexamethasone-treated MSCs (data not shown). Analysis of gene network using Ingenuity indicated that the increased expression of IGF2, IGFBP2 and ITGA5 was inter-related (Fig. 1B). Validation experiments using TaqMan Low-Density Array with two separate experiments using mRNA from two different donors confirmed that IGF2, IGFBP2 and ITGA5 mRNA levels were increased by more than 2-fold in dexamethasone-treated MSCs compared to control cells, thus validating the microarray analysis (Fig. 2A-C). Furthermore, the increased expression of these genes was associated with osteoblast differentiation of hMSCs, as shown by the increased expression of the osteoblast phenotypic genes Runx2, ALP and COL1A1 (Fig. 2D-F). These results indicate that dexamethasone-induced osteoblast differentiation in MSCs is consistently associated with increased expression of IGF2 and IGFBP2 together with ITGA5 in human MSCs.

### IGF2/IGFBP2 promotes human MSCs osteogenic differentiation

**Figure 3:**
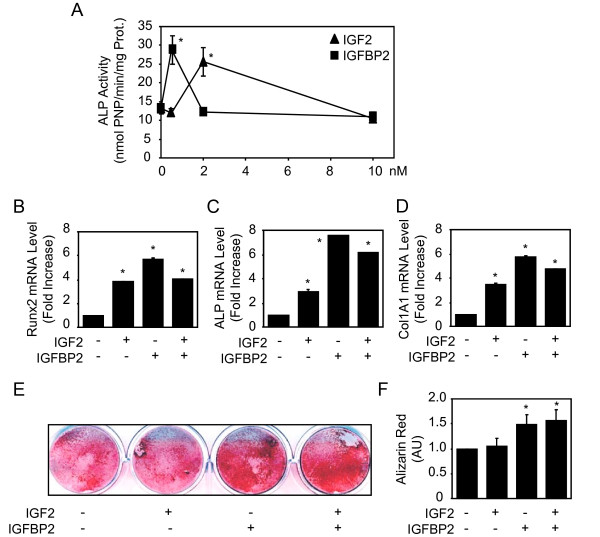
**IGF2/IGFBP2 Promotes Human MSCs Osteogenic Differentiation**. Adult hMSCs were treated with increasing concentrations of IGF2 and IGFBP2 for 4 days and alkaline phosphatase activity (ALP) was determined (**A**). Adult hMSCs were treated with or without IGF2 (2 nM) and IGFBP2 (0.5 nM) for 3 days, total RNA was collected and used for quantitative RT-PCR analysis of Runx2, ALP and Col1A1 expression (**B-D**). Adult hMSCs were cultured in an osteogenic differentiation medium, treated with/without IGF2 (2 nM), IGFBP2 (0.5 nM) or their combination and extracellular matrix mineralization was evaluated by alizarin red staining (**E**) and confirmed by quantification (**F**). Results are expressed as the mean ± SD of treated over control ratio. *: significant difference with untreated cells (*P *< 0.05).

Given the above results, we hypothesized that IGF2 and IGFBP2 may be involved in MSC osteoblast differentiation. To investigate the potential role of these molecules on hMSC osteoblast differentiation, several differentiation assays were conducted. We first analysed the effect of IGF2, IGFBP2 and their combination on ALP activity, an early marker of osteoblast differentiation. A dose dependent effect revealed that IGF2 and IGFBP2 increased ALP activity at the dose of 2 and 0.5 nM, respectively, whereas higher doses had no significant effect in hMSCs (Fig. 3A). We then determined the effects of effective doses of IGF2, IGFBP2 and their combination on osteoblast gene expression. As shown in Fig. 3B-D, we found that IGF2 or IGFBP2 at effective dosages increased Runx2, ALP and COL1A1 mRNA levels in hMSC, though the combination of treatment had no additive effect. To determine whether the increased osteoblast gene expression resulted in increased *in vitro *osteogenesis, human MSCs were cultured in an osteogenic differentiation medium, treated with IGF2, IGFBP2 or their combination and extracellular matrix mineralization was determined. As shown in Fig. 3E, IGFBP2 alone or combined with IGF2 increased extracellular matrix mineralization induced by hMSCs. Quantitative analysis of alizarin red staining confirmed the positive effect of IGFBP2 alone or combined with IGF2 on matrix mineralization (Fig. 3F). Overall, these results show that IGF2 and IGFBP2 have a positive effect on the osteogenic differentiation program in hMSCs.

### ITGA5 Controls IGF2/IGFBP2 expression in hMSCs

**Figure 4:**
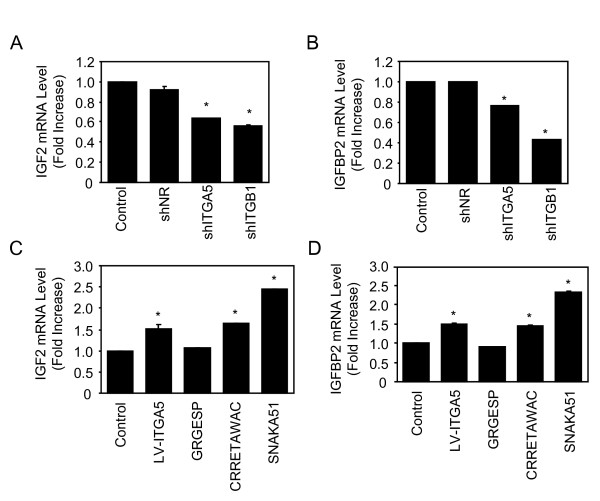
**ITGA5 Controls IGF2/IGFBP2 Expression in hMSCs**. Adult hMSCs were transduced with shITGA5, shITGB1 or a non relevant shRNA (shNR), total RNA was used for quantitative RT-PCR analysis of IGF2 (**A**) and IGFBP2 (**B**). Adult hMSCs were transduced with a lentiviral vector encoding ITGA5, or treated with the agonist peptide CRRETAWAC (100 μg/ml) (CRRETAWAC) or with a non relevant control peptide (GRGESP; 100 μg/ml), or with a conformation-dependent anti-α5 monoclonal antibody (SNAKA51; 10 μg/ml), and IGF2 and IGFBP2 mRNA expression was determined by quantitative RT-PCR analysis (**C-D**). Results are expressed as mean ± SD of treated over control ratio after normalization to 18 S expression. *: significant difference with untreated cells (*P *<0.05).

Our microarray analysis (Fig. 1) revealed that IGF2, IGBP2 and ITGA5 are upregulated during MSC osteoblast differentiation, suggesting possible crosstalks between these molecules. Given our recent data indicate that ITGA5 promotes hMSCs osteoblast differentiation [12], we hypothesized that ITGA5 may act on MSC osteoblast differentiation in part via up-regulation of IGF2 and IGFBP2 in MSCs. To test this hypothesis, we determined whether RNA interference-mediated silencing of ITGA5 expression may blunt IGF2 and IGFBP2 expression in hMSCs. As shown in Figs. 4A and 4B, silencing of ITGA5 expression using an efficient and specific shRNA that decreases ITGA5 mRNA by 50% [12] decreased IGF2 and IGFBP2 mRNA levels in hMSCs, whereas a non relevant control shRNA had no effect. Silencing of integrin β1 (ITGB1) using a specific shRNA also decreased IGF2 and IGFBP2 mRNA levels in hMSCs, supporting a role for the ITGA5/ITGB1 complex in IGF2 and IGFBP2 regulation in hMSCs (Figs. 4A, B). These results indicate that ITGA5 is involved in the regulation of IGF2 and IGFBP2 in hMSCs. To confirm this finding, hMSCs were transduced with a lentiviral vector encoding ITGA5 and IGF2 and IGFBP2 mRNA levels were determined. As shown in figs. 4C and 4D, ITGA5 overexpression in hMSCs increased IGF2 and IGFBP2 mRNA expression, confirming the positive role of ITGA5 in IGF2 and IGFBP2 expression in hMSCs.

We then determined whether activation of endogenous ITGA5 may be sufficient to promote IGF2 and IGFBP2 mRNA expression in hMSCs. To this goal, we used a synthetic peptide (CRRETAWAC) that binds and activates ITGA5 [13]. As shown in Figs. 4C and 4D, the agonist peptide CRRETAWAC (100 μg/ml) markedly increased IGF2 and IGFBP2 mRNA levels in hMSCs whereas a non relevant control peptide (GRGESP; 100 μg/ml) had no effect. To confirm that activation of ITGA5 is sufficient to promote IGF2 and IGFBP2 expresssion, hMSCs were treated with a conformation-dependent anti-α5 monoclonal antibody (SNAKA51; 10 μg/ml) that stimulates α5β1 integrin [13]. As shown in Figs. 4C and 4D, priming ITGA5 using SNAKA51 increased IGF2 and IGFBP2 expresssion in hMSCs. These results provide evidence that ITGA5 controls the expression of IGF2 and IGFBP2, and that activation of ITGA5 using a specific monoclonal antibody or a peptide agonist that primes this integrin is sufficient to promote the expression of IGF2 and IGFBP2 expresssion in hMSCs.

### Inhibition of ITGA5-induced signalling abrogates IGF2/IGFBP2 expression in hMSCs

**Figure 5:**
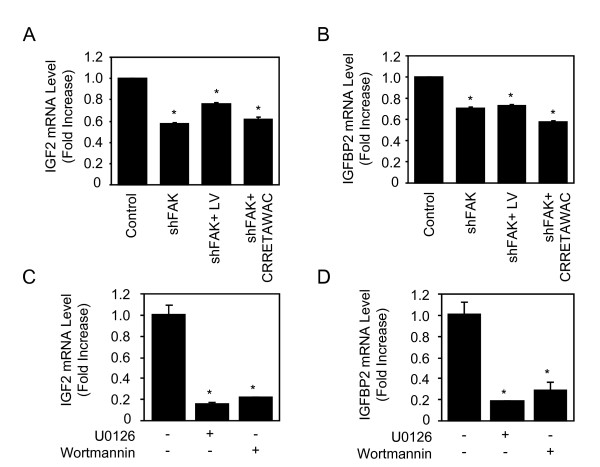
**Inhibition of ITGA5-Induced Signalling Abrogates IGF2/IGFBP2 Expression in hMSCs**. Adult hMSCs transduced with a lentiviral vector encoding ITGA5 or treated with the agonist peptide (CRRETAWAC; 100 μg/ml), and control cells were transiently transfected with a specific shRNA targeting FAK. Total RNA was collected and used for quantitative RT-PCR analysis of IGF2 (**A**) and IGFBP2 (**B**). Adult hMSCs were treated with the MEK inhibitor U0126 (10 μM), or the PI3K inhibitor wortmannin (50 nM) for 24 hours and IGF2 and IGFBP2 mRNA levels were determined by quantitative RT-PCR analysis (**C-D**). Results are expressed as mean ± SD of treated over control ratio after normalization to 18 S expression. *: significant difference with untreated cells (*P *<0.05).

We then determined the signalling mechanisms underlying the promoting effect of ITGA5 on IGF2 and IGFBP2 expression in hMSCs. Integrins are known to activate various signalling pathways, including FAK [14]. We thus hypothesized that FAK and downstream signalling pathways may mediate the increased IGF2 and IGFBP2 expresssion induced by ITGA5 in hMSCs. To test this hypothesis, hMSCs were transfected with a specific shRNA that effectively decreases FAK protein level by 60% [12]. As shown in Figs. 5A and 5B, the expresssion of IGF2 and IGFBP2 mRNA was decreased in shFAK-transfected cells. Furthermore, FAK silencing abolished IGF2 and IGFBP2 expression induced by LV-ITGA5 or CRRETAWAC (Figs. 5A and 5B). These results reveal a key role for FAK signaling in ITGA5-induced IGF2 and IGFBP2 expression in hMSCs. To further investigate the role of downstream pathways, hMSCs were treated with the MEK inhibitor U0126, or wortmannin, a specific PI3K inhibitor. As shown in Figs. 5C and 5D, pharmacological inhibition of MEK or PI3K markedly decreased IGF2 and IGFBP2 expresssion in hMSC. These results indicate that these pathways act downstream of ITGA5/FAK signalling to regulate IGF2 and IGFBP2 expression in hMSCs.

**Figure 6:**
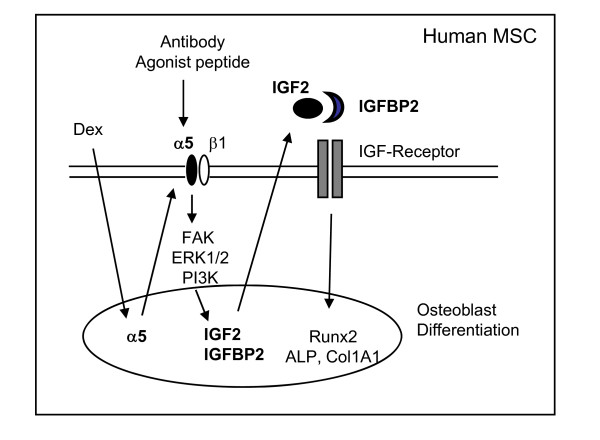
**Proposed Model by which Crosstalks Between ITGA5 and IGF2/IGFBP2 Signalling Trigger Osteogenic Differentiation in Human MSCs**. ITGA5 expression induced by dexamethasone or ITGA5 activation by agonists upregulate signalling pathways that activate IGF2/IGFBP2 expression in hMSCs which in turn triggers osteoblast differentiation, revealing that ITGA5 and IGF2/IGFBP2 crosstalks are important mechanisms controlling osteogenic differentiation in human bone marrow derived mesenchymal stromal cells.

Overall, the results support a mechanism by which dexamethasone-induced ITGA5 expression, or activation of ITGA5 signalling promote IGF2 and IGFBP2 expression which thereby results in activation of osteoblast gene expression in human primary MSCs. This reveals that novel crosstalks between these signalling molecules are important mechanisms that trigger osteogenic differentiation in human mesenchymal stromal cells (Fig. 6).

## Discussion

Finding mechanisms that trigger osteogenic differentiation of hMSCs may help developing novel therapeutic approaches to promote bone formation. In this study, we demonstrate that previously unidentified crosstalks between ITGA5, IGF2 and IGFBP2 are functionally involved in osteoblast differentiation of human MSCs. We first established by microarray analysis that dexamethasone-induced osteoblast differentiation in hMSCs is associated with up-regulation of IGF2 and IGFBP2, suggesting that these molecules are involved in dexamethasone-induced osteoblast differentiation [15]. IGF2 was also found to be up-regulated in dental stem cells during osteogenic differentiation [16], and similar gene expression profiles of IGF2 and BMP2 were reported in differentiated dental follicle cells [17], suggesting an important role of IGF2 in osteogenic differentiation. However, the actions of IGFs and IGFBPs on osteoblastic cells are complex [18,19]. Both IGFs and IGFBPs are expressed by osteoblasts *in vivo *and *in vitro *[20,21]. IGF2 was found to promote ALP activity or collagen synthesis in differentiated osteoblasts [18,22] and IGFBP2 levels were shown to increase during osteoblast differentiation [23]. However, little is known on the role of IGF2 and IGFBP2 in the early stages of osteoblastogenesis. We show here that IGF2 and IGFBP2 are functionally involved in the osteogenic differentiation of human mesenchymal stromal cells. Indeed, we found that IGF2/IGFBP2 at low doses increased the expression of osteoblast phenotypic genes and *in vitro *osteogenic capacity of hMSCs. The previously unrecognised positive role of IGF2/IGFBP2 on osteogenic differentiation of hMSCs *in vitro *suggests that these molecules may be anabolic *in vivo*. This is supported by the finding that subcutaneous administration of IGF2/IGFBP2 stimulates bone formation and prevents bone loss in osteopenic rats [24]. A positive role for IGFBP2 in bone formation and trabecular bone acquisition has also been reported in mice [25]. This anabolic effect is consistent with the recent finding that IGF2 and IGFBP2 levels and bone formation are increased in patients with osteosclerosis linked to hepatitis C [26]. The present study revealing that IGF2/IGFBP2 triggers human MSC osteoblast differentiation provides one cellular mechanism that may account for the positive effect of IGF2/IGFBP2 on osteoblastogenesis and bone formation.

Several lines of evidence point to functional links between IGF2 and IGFBP2 and ITGA5 in hMSCs. First, we found that ITGA5 silencing reduced IGF2 and IGFBP2 expression. Second, we showed that ITGA5 overexpression up-regulated IGF2 and IGFBP2, indicating that ITGA5 acts upstream of IGF2 and IGFBP2 in hMSCs. Third, specific activation of ITGA5 using an anti-ITGA5 monoclonal antibody that primes the integrin, or a peptide that acts as an ITGA5 agonist promoted IGF2 and IGFBP2 expression in hMSCs, further demonstrating that ITGA5 signalling plays a key role in the regulation of IGF2 and IGFBP2 in hMSCs. Integrins have been previously found to interact with IGFBP2 in cancer cells [27,28]. Whether ITGA5 may directly interact with IGFBP2 to trigger cell signalling and osteogenic differentiation in hMSCs is an interesting possibility that remains to be determined. One mechanism by which ITGA5 may activate IGF2 and IGFBP2 expression in hMSCs is via activation of signalling pathways that are activated by ITGA5. Interestingly, our data showed that FAK, ERK1/2 and PI3K that are all activated by ITGA5 [29] mediate the increased IGF2 and IGFBP2 expression in hMSCs. These data provide novel mechanisms by which activation of ITGA5 and downstream FAK, ERK1/2 and PI3K signalling upregulates autocrine IGF2/IGFBP2 expression and thereby promotes osteoblast differentiation in human mesenchymal stromal cells.

## Conclusions

The present study identifies a sequence of molecular mechanisms by which ITGA5 expression or activation and downstream activation of FAK, ERK1/2 and PI3K signalling induce IGF2/IGFBP2 expression in hMSCs. Furthermore, this study reveals novel crosstalks between ITGA5 and IGF2/IGFBP2 signalling that converge to induce osteoblast differentiation in hMSCs (Fig. 6). These mechanisms may help designing novel therapeutic strategies for promoting the osteogenic differentiation potential of human mesenchymal stromal cells.

## Methods

### Mesenchymal Stromal Cells

Human primary mesenchymal stromal cells (hMSCs) were obtained from bone marrow aspirates (iliac crest) of patients undergoing hip replacement surgery. Informed consent from each patient was obtained before surgery [30] according to the French regulation. Cells were cultured in Dulbecco's Modified Eagle's Medium (DMEM; Invitrogen Corporation, Paisley, Scotland) supplemented with 10% heat inactivated Fetal Calf Serum (FCS; PAA Laboratories, Les Mureaux, France), L-glutamine (292 mg/l) and antibiotics (10,000 U/ml penicillin and 10,000 μg/ml streptomycin) at 37°C in a humidified atmosphere containing 5% CO2 in air. Cells were expanded and used at passage 2.

### Materials

The lentiviral vector encoding complete sequence of ITGA5 was obtained as previously described [29]. Two different shRNA species were tested. The data shown are representative of the most effective shRNA. Non relevant shRNA (scrambled sequence that does not lead to specific degradation of any known cellular mRNA), ITGB1 shRNA and human FAK shRNA lentiviral particles (mixtures of viral particles containing 3 target-specific constructs that encode shRNA designed to knock down gene expression) were obtained from Santa Cruz Biotechnology (Heidelberg, Germany). Western blot analysis showed that gene expression was effectively down-regulated using these gene specific shRNAs (12). The ITGA5 activating monoclonal antibody (SNAKA51) and the synthetic agonist peptide (CRRETAWAC) were kindly provided by Dr. M.J. Humphries (University of Manchester, UK). A rabbit IgG fraction (negative control) was from Dako (Trappes, France). The synthetic control peptide (GRGESP, used as control for CRRETAWAC in USA Patent Number 5,627,263 by E. Ruoslahti and E. Koivunen, 1997) was from Peptide 2.0 Inc (Chantilly, VA, USA). The pharmacological inhibitors U0126 and Wortmannin were from Sigma-Aldrich (St. Louis, MI, USA).

### Microarray Analysis

Subconfluent human MSCs were incubated with or without 10^-7 ^M dexamethasone for 1, 3 or 7 days, and total RNA was isolated using an RNeasy kit (Qiagen, Courtaboeuf, France) according to the manufacturer's recommendations. Gene expression profiling with Affymetrix HG-U133 Plus 2.0 arrays was performed in three different studies using primary human MSCs from different donors as described in our previous original study [11]. Briefly, cDNA was synthesized from 1 μg of total RNA and transcribed into biotin-labelled cRNA. Fifteen micrograms of fragmented cRNA per 300 μl were then hybridized to the GeneChips for 16 h at 45°C. Hybridization was performed according to the standard Affymetrix protocol. GeneChips were washed, stained and scanned with the GeneArray scanner controlled by Affymetrix GCOS software. Raw gene expression data were processed with the GCOS 1.2 software for signal calculation. Comparison analysis and cellular pathways were analyzed using statistical tools (http://www.bioretis.de; http://www.ingenuity.com/index.html). The microarray data are available on line http://www.ncbi.nlm.nih.gov/geo/query/acc.cgi?acc=GSE18043.

### Quantitative RT-PCR Analyses

Total RNA was isolated using RNeasy Kit (Qiagen) according to the manufacturer's recommendations. cDNA synthesis was performed using the High Capacity cDNA Reverse Transcription kit (Applied Biosystems). Each reaction containing 3 μg of total RNA, 1× RT buffer, 1 mM dNTP mix, 1× random primers and 50 U multiscribe reverse transcriptase in a total volume of 100 μl. The reverse transcription reactions were run under the following conditions: 25°C for 5 min, 37°C for 120 min and 95°C for 2 minutes. Products of reverse transcription were analysed either on a predesigned TaqMan Low-Density Array (sets for 47 human genes and GAPDH RNA as a reference gene) based on an Applied Biosystems 7900HT 384-well format Microfluidic Card, or by quantitative PCR using LightCycler (Roche Applied Science, Indianapolis Ind., USA) and SYBR Green PCR kit (ABGen, Courtabœuf, France), as previously described [29].

### Alkaline Phosphatase Activity

Cells were lyzed and sonicated in ice cold H_2_O. Lysates were centrifuged at 3500 rpm for 15 min at 4°C and ALP activity was determined using the Alkaline Phosphatase kit (BioRad; Hercules, USA) as previously described [31]. Total protein content was determined using BioRad reagent (BioRad). In parallel, cell monolayers were fixed in ethanol (70%) before ALP activity cytochemical detection using Sigma FAST BCIP/NBT kit. Wells were microphotographed using an Olympus microsocope Japan).

### Osteogenic Assay

For *in vitro *matrix mineralization, the medium was supplemented with ascorbic acid (50 μg/ml) and inorganic phosphate (NaH_2_PO_4_; 3 mM) to induce collagenous matrix synthesis and mineralization [32]. After 14 days of culture, cells were fixed in 4% paraformaldehyde in PBS. Matrix mineralization was detected by alizarin red staining (40 mM, pH 4.2) and microphotographed using an Olympus microsocope (Japan). Alizarin red content was quantified spectrophotometrically after dissolution in cetylpyridinium chloride (10% in PBS).

### Statistical Analysis

The results are expressed as mean ± SD of at least 3 samples. Comparisons between data were performed using the two-factor analysis of variance (ANOVA) using the statistical package super-ANOVA (Macintosh, Abacus concepts, Inc., Berkeley, CA). A minimal level of *P *< 0.05 was considered significant.

## Abbreviations

(ALP): Alkaline phosphatase; (Col1A1): Type I collagen; (ERK1/2): Extracellular-related kinases 1 and 2; (FAK): Focal adhesion kinase; (ITGA5): Integrin alpha 5; (ITGB1): Integrin beta I; (MAPK): Mitogen-activated protein kinases; (MSCs): Mesenchymal stromal cells; (PI3K): Phosphatidylinostol 3-kinase.

## Authors' contributions

ZH and OF participated in the design of the study, carried out the experiments, analysed the data and drafted part of the manuscript. JR and TH carried out the microarray analysis. PJM conceived the study, participated in its design and coordination and wrote the manuscript. All authors read and approved the final manuscript.
